# Synergistic effects of various Her inhibitors in combination with IGF-1R, C-MET and Src targeting agents in breast cancer cell lines

**DOI:** 10.1038/s41598-017-04301-8

**Published:** 2017-06-21

**Authors:** Aryan Stanley, G. Hossein Ashrafi, Alan M. Seddon, Helmout Modjtahedi

**Affiliations:** Kingston University London, School of Life Science, London, United Kingdom

## Abstract

Overexpression of HER2 has been reported in around 25% of human breast cancers. Despite recent advances in HER2 targeted therapy, many patients still experience primary and secondary resistance to such treatments, the mechanisms for which are poorly understood. Here, we investigated the sensitivity of a panel of breast cancer cell lines to treatment with various types of HER-family inhibitors alone or in combination with other tyrosine kinase inhibitors or chemotherapeutic agents. We found that treatment with the second-generation irreversible HER-family inhibitors, particularly afatinib and neratinib, were more effective than treatment with the first-generation reversible inhibitors in inhibiting growth, migration and downstream cell signalling in breast cancer cells. Of the three HER2 overexpressing cell lines in this panel, SKBr3 and BT474 were highly sensitive to treatment with HER-family inhibitors, while MDA-MB-453 was comparatively resistant. Combinations of HER-family inhibitors with NVP-AEW541, dasatinib or crizotinib (inhibitors of IGF-1R, Src and c-Met/ALK, respectively) led to synergistic effects in some of the cell lines examined. In particular, treatment with a combination of Src and HER-family member inhibitors resulted in synergistic growth inhibition of MDA-MB453 cells, implicating Src as a mediator of resistance to HER2-targeting agents. Our results suggest that combining HER-family inhibitors with other TKIs such as dasatinib may have therapeutic advantages in certain breast cancer subtypes and warrants further investigation.

## Introduction

Despite significant advances in diagnosis and treatment in recent years, breast cancer is still the most commonly diagnosed cancer among women worldwide, with over 1.6 million cases (accounting for 25% of all cancers) diagnosed in 2012^1^. Breast cancer also has the highest mortality of any cancer in women worldwide^1^ and the second highest in the United Kingdom^2^. Major challenges in breast cancer management are primary or acquired resistance to current therapies. These in turn underline the need for further research to develop a better understanding of the mechanisms of resistance to therapy and for development of more effective therapeutic and less toxic approaches for the management of breast cancer^3–5^.

The Human Epidermal Growth Factor Receptor (HER) family is a well characterised group of membrane-bound receptor tyrosine kinases (RTKs) which consists of four closely related members: EGFR (HER1), HER2, HER3 and HER4^6–8^. The binding of HER ligands to the extracellular domain of the receptor leads to homo- or hetero-dimerisation of the HER family, the activation of downstream signalling pathways, such as the *ras-raf-*mitogen-activated protein kinase (MAPK) pathway (associated with proliferation) and the phosphatidylinositol 3 kinase protein (PI3K)/Akt pathway (associated with cell survival). Deregulation of these pathways due to aberrant expression or activation of HER-family members can lead to increased proliferation, reduced apoptosis, angiogenesis and invasiveness, which are the hallmarks of cancer. As such, HER family members have become popular targets for therapy, specifically by small molecule tyrosine kinase inhibitors (TKIs) and monoclonal antibodies (mAbs)^8–10^.

Overexpression of HER-2 has been reported in around 25% of breast cancer patients and has been associated with a poorer prognosis in many such patients^11–14^. Several therapies have been approved for the treatment of HER2-positive (HER2+) breast cancers: Lapatinib, a dual-targeting (EGFR/HER2) reversible TKI; Trastuzumab (Hercepin), a humanised mAb that binds to domain IV of the HER2 receptor; Pertuzumab (Perjeta) another humanised mAb that binds to domain II of the HER2 receptor; and Trastuzumab emtansine (Kadcyla or T-DM1), a conjugate of Trastuzumab and the cytotoxic drug DM1^15–17^. Despite these treatments improving overall progression free survival (PFS) and clinical benefit rate (CBR), not all patients with HER2 + tumours respond to targeted therapy and many that do often relapse^15, 18–21^. It is therefore essential to develop new and more effective treatments to overcome resistance to therapy, and to discover novel predictive biomarkers for use in the selection of patients who may benefit from such therapeutic inventions.

A number of other tyrosine kinase proteins commonly associated with cancer, such as IGF-1R, c-Met and Src, have been shown to co-operate with HER-family members to drive tumour growth and confer resistance to treatments. For example, increases in expression or signalling of IGF-1R, a RTK also involved in the regulation of cell growth, differentiation and survival, has been linked to resistance against HER2 targeted therapies, particularly trastuzumab, possibly via IGF-1R/HER2 hetero-dimerisation^13, 22–24^. In other studies, activation of c-Met, another RTK involved in cell migration, invasion, survival and angiogenesis, has been shown to contribute to primary trastuzumab resistance, and upregulation of c-Met has been associated with secondary resistance to trastuzumab in HER2-overexpressing cells^25, 26^. While c-Met activation has been shown to mediate resistance to lapatinib in gastric cancer cells^27–29^, the role of c-Met in resistance to HER-family inhibitors in breast cancer has been less extensively studied. In other studies, expression of members of the Src family, a group of non-receptor tyrosine kinases, has been correlated with increased expression of HER-family members^30–32^. Strong positive associations have also been made between Src expression and increased HER2/HER3 dimerisation, including the possibility that Src slows the rate of internalisation of HER2/HER3 hetero-dimers, prolonging their downstream signal activation^33, 34^. Due to their co-operative interactions, co-targeting of these tyrosine kinases alongside HER-family members when appropriate may lead to synergistic effects and improved clinical efficacy over single agents.

The aim of this study was to investigate the sensitivity of a panel of human breast cancer cell lines to treatment with various reversible and irreversible HER-family inhibitors, alone and in combination with other treatments including an IGF-1R inhibitor (NVP-AEW541), a c-Met/ALK inhibitor (crizotinib), an Abl-Bcr/c-Kit inhibitor (imatinib), an Abl-Bcr/Src family inhibitor (dasatinib) and the chemotherapeutic agents paclitaxel and gemcitabine. We then investigated whether there was an association between receptor expression and sensitivity to treatment. In addition, we investigated the effect of selected TKIs on the phosphorylation of HER receptors and the downstream molecules, MAPK and Akt, cell cycle distribution and migration of breast cancer cells.

## Results

### HER2 is overexpressed in BT474, SKBr3 and MDA-MB-453 while EGFR is overexpressed in MDA-MB-468

**Figure 1:**
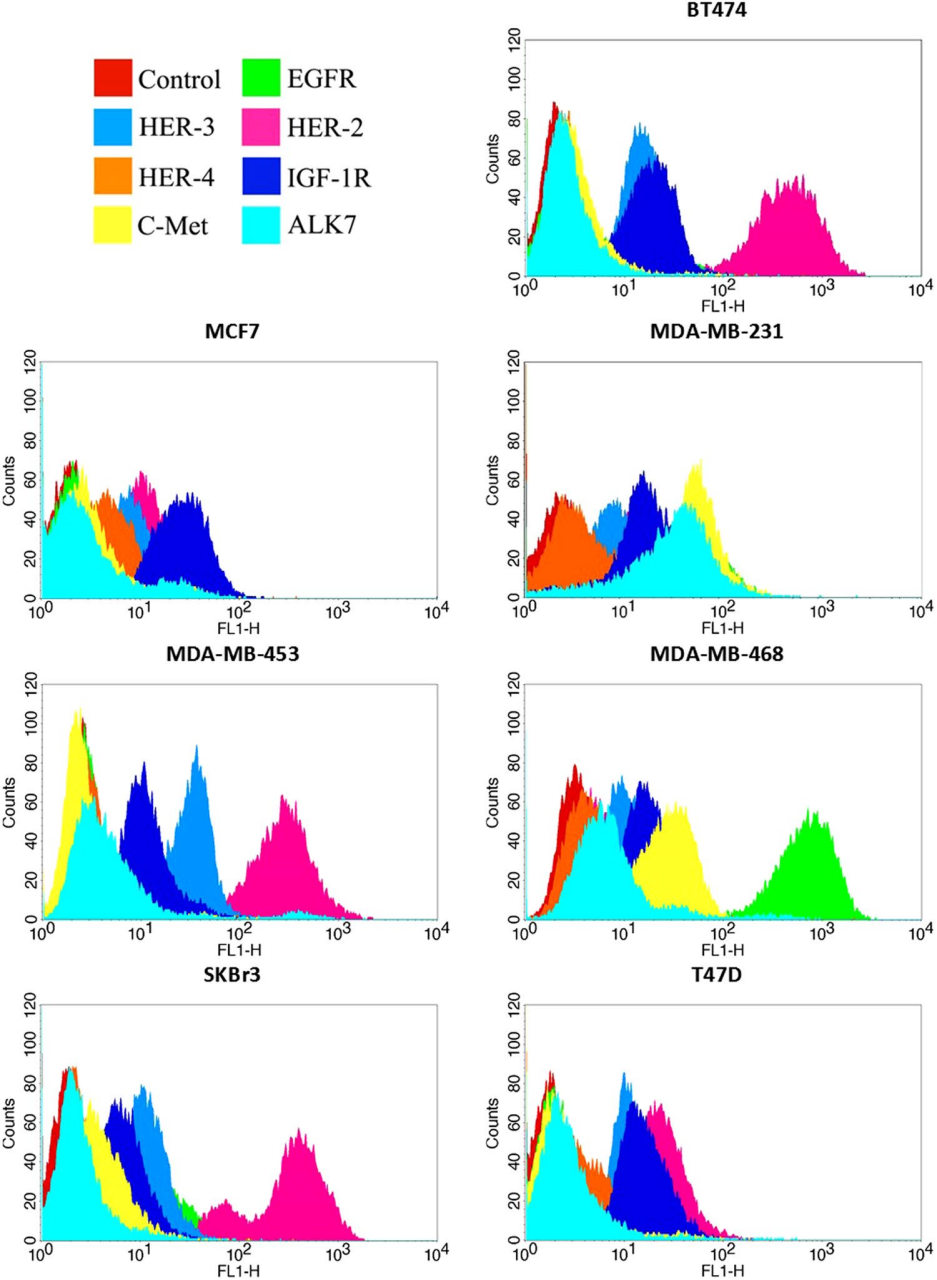
The cell surface expression of growth factor receptors determined by flow cytometry in human breast cancer cell lines represented as histograms, as described in the materials and method section. Histograms are plotted as number of events (cells counted) against forward light scatter (FITC)

Using flow cytometry, we determined the membrane expression levels of all four HER-family members, IGF-1R, c-Met and ALK in our panel of breast cancer cell lines, with expression being represented as mean fluorescence intensity (MFI) (Table 1, Fig. 1). Of the seven cell lines tested, one had a very high expression of EGFR (MDA-MB-468, MFI = 799.43) while three had high expressions of HER2 (BT474, MFI = 540.81; MDA-MB-453, MFI = 315.56; SKBr3, MFI = 467.53). All other HER-family receptors had barely detectable to moderate expressions in this panel. Of note, EGFR and HER3 were expressed at levels above 30 MFI in MDA-MB-231 and MDA-MB-453, respectively. HER4 expression was barely detectable in any cell line. IGF-1R had low to moderate expression in our panel of cell lines, MCF7 having the highest (MFI = 28.26). C-Met expression ranged from barely detectable to moderate, MDA-MB-231 and MDA-MB-468 having the highest (MFI = 26.50 and 31.20, respectively). ALK expression was barely detectable in any cell line except MDA-MB-231, which had moderate expression (MFI = 21.24).

**Table 1:** Expression of RTKs in breast cancer cell lines assessed by Flow Cytometry.

Mean Fluorescence Intensity (MFI)								
Cell Line	Control	EGFR	HER2	HER3	HER4	IGF-1R	C-Met	ALK
BT474	2.3 ± 0.4	2.8 ± 0.6	540.8 ± 105.1	16.7 ± 2.2	2.8 ± 0.3	18.8 ± 2.7	3.3 ± 0.5	2.6 ± 0.4
MCF7	2.1 ± 0.4	2.2 ± 0.4	12.1 ± 2.2	8.9 ± 1.6	5.1 ± 0.9	28.3 ± 6.0	2.7 ± 0.5	2.3 ± 0.4
MDA-MB-231	4.2 ± 0.5	37.7 ± 5.9	21.8 ± 3.3	6.5 ± 1.0	8.4 ± 1.2	21.0 ± 3.2	26.5 ± 4.3	21.2 ± 3.4
MDA-MB-453	2.7 ± 0.4	2.9 ± 0.4	315.6 ± 50.5	32.7 ± 5.1	3.1 ± 0.5	11.4 ± 1.8	2.4 ± 0.4	4.3 ± 0.7
MDA-MB-468	3.8 ± 0.5	799.4 ± 111.3	5.1 ± 0.7	11.0 ± 1.6	4.3 ± 0.6	18.5 ± 2.7	31.2 ± 4.5	6.3 ± 0.9
SKBr3	2.0 ± 0.3	12.5 ± 2.4	467.5 ± 90.5	12.5 ± 2.2	2.6 ± 0.4	7.2 ± 1.4	4.3 ± 0.7	2.4 ± 0.4
T47D	2.0 ± 0.3	2.3 ± 0.3	26.8 ± 3.6	13.6 ± 1.9	4.2 ± 0.6	17.2 ± 2.5	2.3 ± 0.3	2.7 ± 0.4

The data is presented as the mean fluorescence intensity (MFI) ± Standard Deviation (SD) of gated events

Irreversible HER-family TKIs are highly effective at inhibiting growth of HER2 overexpressing cell lines SKBr3 and BT474, but not MDA-MB-453.

**Figure 2:**
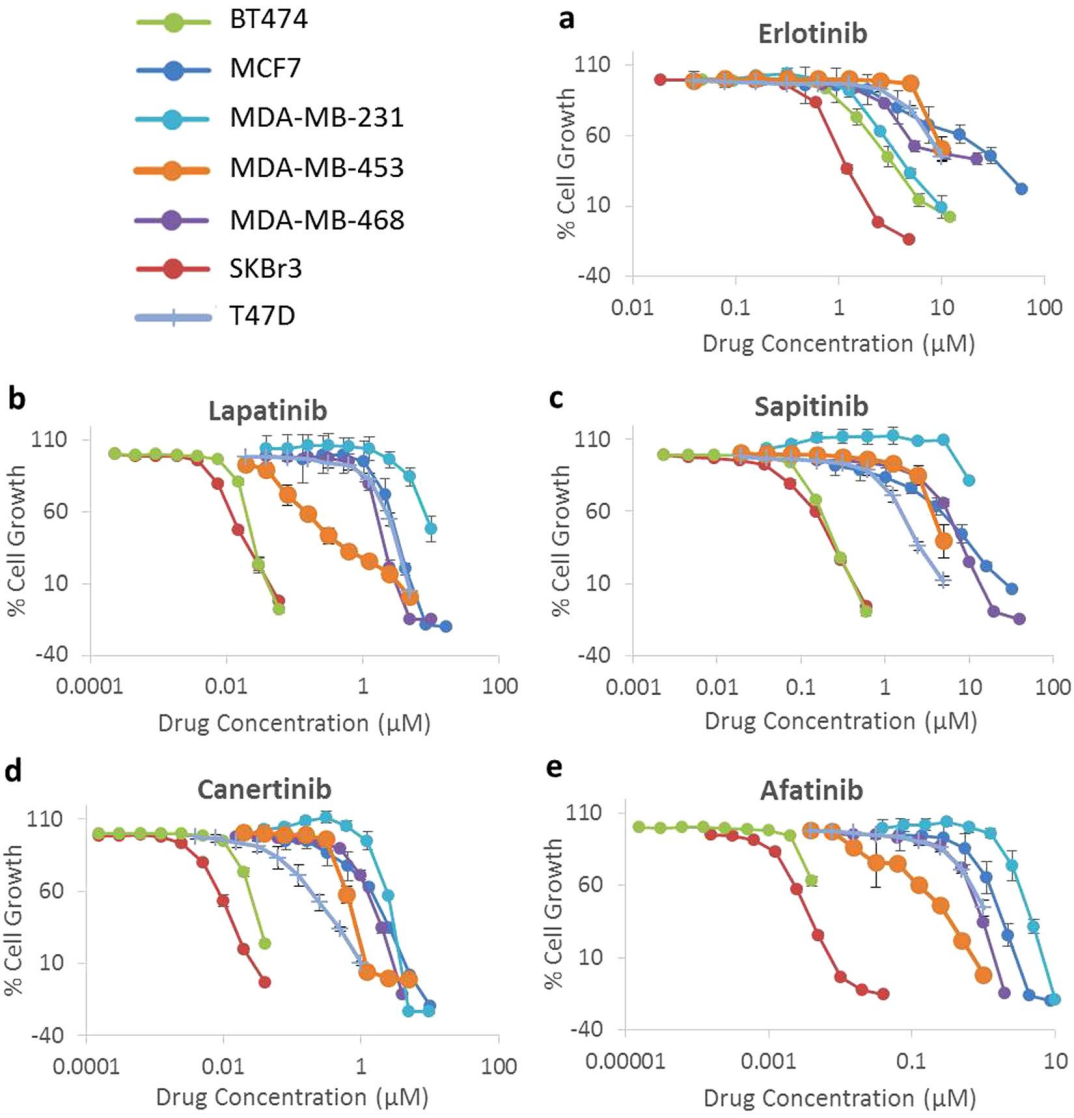
Growth control graphs showing effect of selected HER-family TKIs in doubling concentrations on growth of breast cancer cell lines. (**a**) EGFR reversible inhibitor erlotinib. (**b**) Dual EGFR/HER2 reversible inhibitor lapatinib. (**c**) Pan-HER reversible inhibitor sapitinib. (**d**) Pan-HER irreversible inhibitor canertinib. (**e**) Pan-HER irreversible inhibitor afatinib. Sulforhodamine B colorimetric assay was used to determine the effect of treatment of breast cancer cell lines with doubling dilutions of HER-family inhibiting TKIs. The irreversible pan-inhibitors (e.g. afatinib, canertinib, neratinib) were consistently more effective than the reversible dual and pan inhibitors (e.g. lapatinib, sapitinib), which were in turn more effective than the reversible EGFR inhibitors (e.g. erlotinib, gefitinib). Each point is a representative of the mean ± SD of triplicate samples.

We investigated the growth response of our panel of breast cancer cell lines to treatment with various kinase inhibitors and chemotherapeutic agents using the SRB assay (Table 2). The HER2 overexpressing cell lines BT474 and SKBr3 were most susceptible to treatment with HER family targeting TKIs, with IC50s as low as 3-4 nM for afatinib and neratinib. The HER2 overexpressing cell line MDA-MB-453 was, by comparison, significantly more resistant to treatment with the same drugs, with IC50s of 0.11 µM and 0.13 µM for neratinib and afatinib, respectively. Generally, the irreversible inhibitors (afatinib, neratinib, canertinib) were more effective at inhibiting cell growth than their reversible counterparts (Fig. 2).

**Table 2:** IC50 values for cell lines treated with various agents. (a) HER-family targeting TKIs and (b) other TKIs and chemotherapeutic agents.

a	IC_50_ Values (µM)						
Cell Line	Erlotinib	Gefitinib	Lapatinib	Sapitinib	Afatinib	Neratinib	Canertinib
BT474	2.88	0.46	0.023	0.24	0.004	0.003	0.031
MCF7	>10	>10	2.66	6.73	1.55	0.35	1.60
MDA-MB-231	3.28	>10	9.29	>10	3.69	1.35	2.59
MDA-MB-453	>10	7.35	0.26	4.37	0.13	0.11	0.67
MDA-MB-468	5.71	1.73	1.73	6.57	0.82	0.036	1.51
SKBr3	1.06	4.50	0.015	0.20	0.003	0.003	0.011
T47D	9.21	8.82	2.87	1.91	0.89	0.86	1.41
**b**	**IC**_**50**_ **Values (µM)**						
**Cell Line**	**NVP-AEW541**	**Dasatinib**	**Imatinib**	**Crizotinib**	**Paclitaxel**	**Gemcitabine**	
BT474	2.29	0.11	9.20	2.30	0.007	0.047	
MCF7	0.14	4.95	>10	0.42	0.002	0.058	
MDA-MB-231	2.84	0.009	9.46	1.09	0.024	1.389	
MDA-MB-453	1.91	2.79	8.94	0.40	0.002	0.004	
MDA-MB-468	1.57	0.087	5.44	0.76	0.003	0.003	
SKBr3	3.80	4.17	9.45	4.61	0.002	0.35	
T47D	5.27	0.067	8.38	1.16	0.001	0.38	

Each value is the mean of triplicate samples.

**Figure 3:**
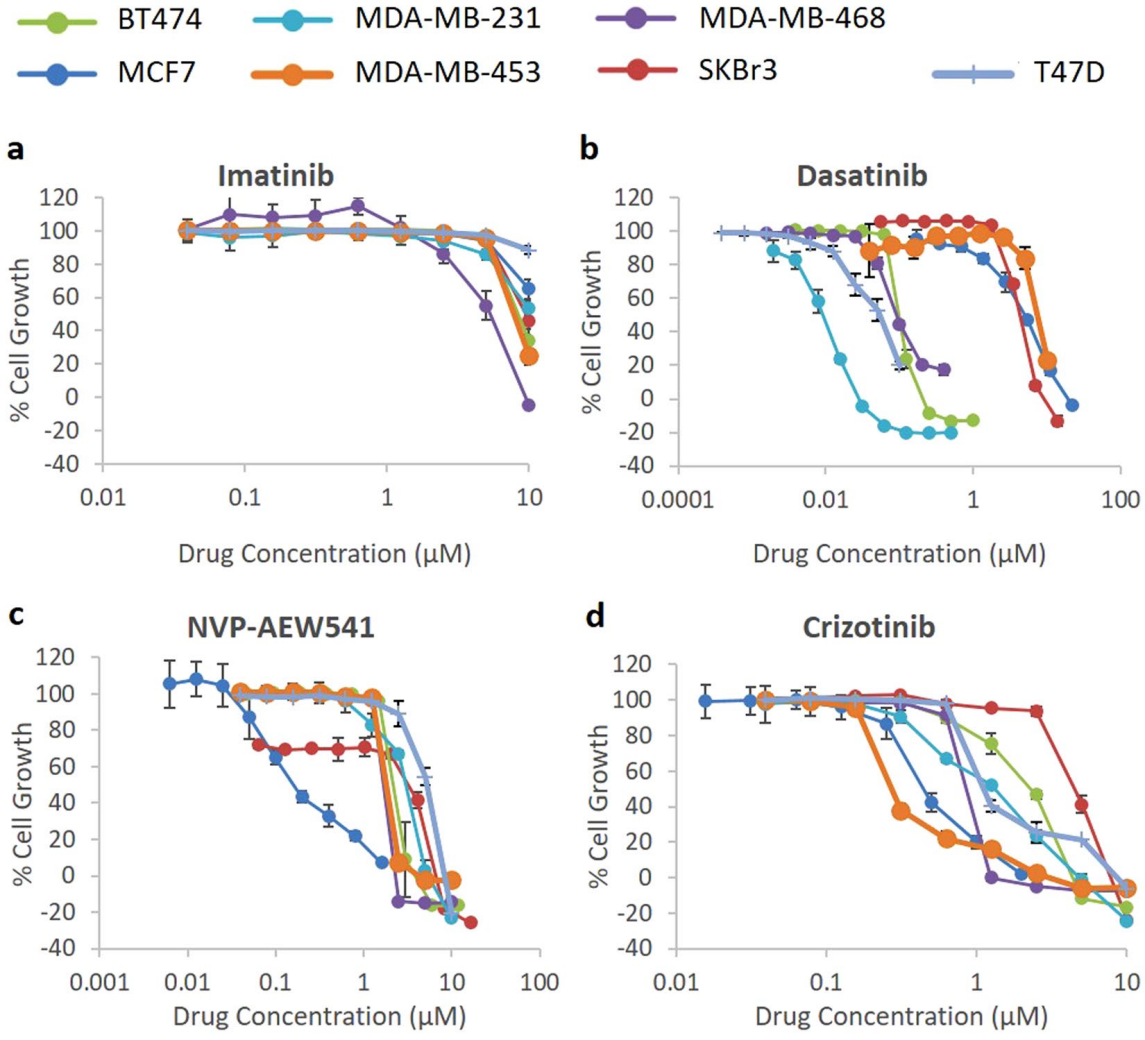
Growth control graphs showing effect of selected TKIs in doubling concentrations on growth of breast cancer cell lines. (**a**) BCR-Abl/c-Kit inhibitor imatinib. (**b**) BCR-Abl/Src inhibitor dastinib. (**c**) IGF-1R inhibitor NVP-AEW541. (**d**) C-Met/ALK inhibitor crizotinib. Sulforhodamine B colorimetric assay was used to determine the effect of treatment of breast cancer cell lines with doubling dilutions of selected TKIs. Imatinib was relatively ineffectual at inhibiting growth in our panel of cell lines, while the effects of dasatinib ranged from moderate (MCF7, IC50 = 4.95 µM) to very high (MDA-MB-231, IC50 = 9 nM). NVP-AEW541 and Crizotinib had moderate growth inhibitory effects in all cell lines, ranging from 0.14–5.27 µM and 0.40–4.61 µM, respectively. Each point is a representative of the mean ± SD of triplicate samples.

While the Abl-Bcr/c-Kit inhibitor imatinib had poor growth inhibitory effects on all cell lines in our panel (IC50 range 5.44 µM to > 10 µM), the Abl-Bcr/Src inhibitor dasatinib was considerably more effective, with IC50 values as low as 9 nM (MDA-MB-231) (Table 2, Fig. 3). The IGF-1R inhibitor NVP-AEW541 and the c-Met/ALK inhibitor crizotinib moderately inhibited growth of all cell lines.

**Figure 4:**
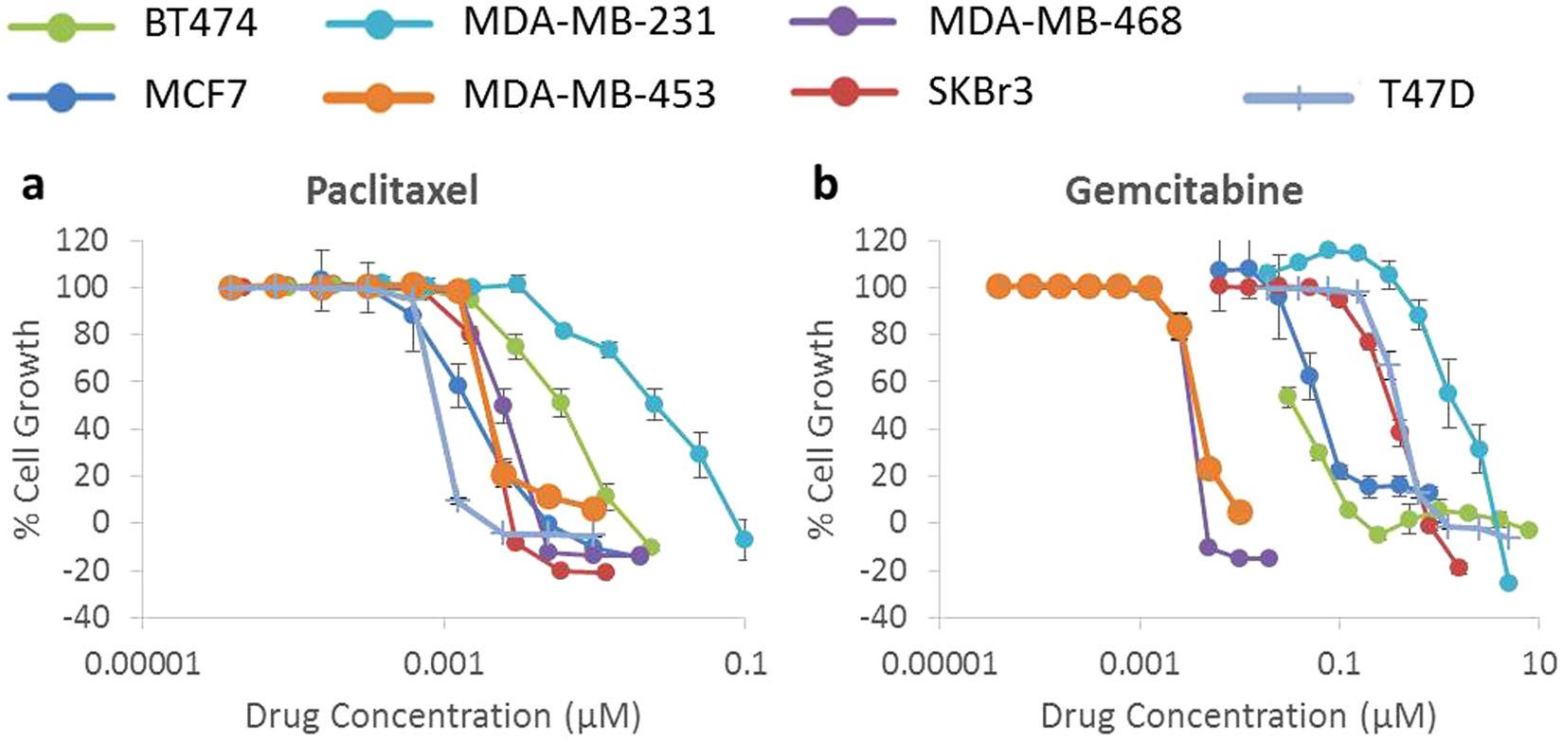
Growth control graphs showing effect of chemotherapeutic drugs paclitaxel and gemcitabine in doubling concentrations on growth of breast cancer cell lines. (**a**) Chemotherapy taxane paclitaxel. (**b**) Chemotherapy nucleoside analog gemcitabine. Paclitaxel was highly effective at inhibiting growth in all breast cancer cell lines, with IC50s ranging from 1 nM (T47D) to 24 nM (MDA-MB-231). Gemcitabine had moderate to high effects, with IC50s ranging from 3 nM (MDA-MB-468) to 1.389 µM (MDA-MB-231). Each point is a representative of the mean ± SD of triplicate samples.

Paclitaxel was very effective in all cell lines, with IC50s ranging from 1 nM (T47D) to 24 nM (MDA-MB-231), while gemcitabine was generally less effective, with IC50s ranging from 3 nM (MDA-MB-468) to 1.39 µM (MDA-MB-231) (Table 2, Fig. 4). MDA-MB-231 was the most resistant to these chemotherapy drugs.

### Treatment of human breast cancer cell lines with combinations of selected TKIs or chemotherapeutic agents with HER-family inhibitors leads to synergistic effects in some cell lines and antagonistic effects in others

The growth responses of breast cancer cell lines BT474, MCF7, MDA-MB-468, SKBr3 and also MDA-MB-453 to combinations of various inhibitors and cytotoxic drugs were investigated. Median effect analyses showed varying results between cell lines (Table 3). For example, the combination of NVP-AEW541 with HER-family TKIs had synergistic effects on MCF7 and MDA-MB-468, but mixed results in HER2 overexpressing cell lines BT474, SKBr3 and MDA-MB-453, ranging from synergy to moderate antagonism. Dasatinib plus HER-family TKIs were antagonistic in MCF7 and SKBr3, synergistic in MDA-MB-468 and MDA-MB-453, and had mixed effects in BT474. Crizotinib plus HER-family TKIs were mainly synergistic in MCF7 and MDA-MB-468, mostly antagonistic in BT474 and SKBr3, and had mixed results in MDA-MB-453. Paclitaxel plus HER-family TKIs were mainly synergistic in BT474 and MDA-MB-468 and had mixed effects in MCF7 and SKBr3. Gemcitabine plus HER-family inhibitors had antagonistic effects in BT474 and SKBr3 and mixed effects in MCF7 and MDA-MB-468. No set of drug combinations were consistently synergistic or antagonistic in all the cell lines tested.

**Table 3:** Combination Indices for combined agents on cell lines MCF7, SKBr3, BT474, MDA-MB-468 and MDA-MB-453.

Drug Combination	Combination Index Mean (range)					
		MCF7	SKBr3	BT474	MDA-MB-468	MDA-MB-453
NVP+	Erl	0.35 (0.24–0.46)	1.26 (1.02–1.49)	0.64 (0.57–0.70)	0.66 (0.61–0.71)	0.80 (0.58–1.01)
	Lap	0.40 (0.26–0.54)	1.10 (1.01–1.18)	1.44 (1.23–1.64)	0.90 (0.86–0.95)	0.51
	Sap	0.26 (0.18–0.35)	0.80 (0.79–0.81)	1.38 (1.34–1.42)	0.84 (0.79–0.89	0.99 (0.90–1.09)
	Afa	0.50 (0.29–0.71)	0.48 (0.43–0.53)	1.26 (1.00–1.51)	0.80 (0.74–0.87)	0.83 (0.60–1.05)
	Can	0.37 (0.18–0.55)	0.92 (0.91–0.92)	0.81 (0.68–0.93)	0.84 (0.77–0.90)	1.30 (0.91–1.69)
	Das	0.49 (0.38–0.59)	3.47 (3.24–3.70)	1.54 (1.06–2.02)	0.67 (0.61–0.72)	0.47 (0.42–0.51)
	Criz	1.20 (1.14–1.25)	0.88 (0.84–0.90)	1.07 (0.93–1.21)	1.15 (1.13–1.17)	0.71 (0.49–0.93)
	Pac	0.81 (0.73–0.90)	0.84 (0.80–0.89)	1.16 (0.95–1.37)	2.29 (2.22–2.36)	—
	Gem	0.78 (0.60–0.97)	0.96 (0.87–1.05)	1.63 (1.48–1.77)	2.88 (2.66–3.11)	—
Das+	Erl	4.62 (2.89–6.35)	1.87 (1.62–3.11)	0.46 (0.43–0.50)	0.11	0.56 (0.38–0.75)
	Lap	1.23 (1.07–1.40)	1.60 (1.47–1.73)	1.46 (1.01–1.91)	0.46 (0.45–0.46)	0.19 (0.15–0.22)
	Sap	2.90 (1.24–4.55)	1.72 (1.70–1.75)	1.04 (0.91–1.17)	0.42	0.49 (0.27–0.71)
	Afa	3.45 (2.61–4.29)	2.20 (2.16–2.23)	0.78 (0.57–0.99)	0.22 (0.21–0.23)	0.36 (0.29–0.44)
	Can	0.88 (0.74–1.01)	1.83 (1.80–1.87)	0.81 (0.61–1.02)	0.25 (0.24–0.26)	0.60 (0.43–0.77)
	Criz	0.96 (0.95–0.97)	1.45 (1.42–1.48)	0.66 (0.46–0.86)	0.66 (0.65–0.68)	0.53 (0.31–0.75)
	Pac	1.64 (0.79–2.48)	1.48 (1.38–1.58)	0.86 (0.78–0.94)	0.54 (0.44–0.64)	—
	Gem	2.60 (1.79–3.41)	1.50 (1.32–1.68)	0.93 (0.77–1.08)	0.68 (0.66–0.70)	—
Criz+	Erl	0.70 (0.54–0.86)	2.22 (1.48–2.97)	1.07 (1.04–1.11)	0.79 (0.78–0.81)	1.01 (0.70–1.32)
	Lap	0.53 (0.40–0.67)	2.16 (1.95–2.38)	1.43 (1.26–1.60)	0.99 (0.94–1.04)	0.83 (0.54–1.11)
	Sap	0.37 (0.35–0.39)	2.06 (1.44–2.68)	1.47 (1.13–1.80)	1.02 (0.99–1.04)	1.65 (1.48–1.81)
	Afa	0.85 (0.61–1.09)	1.44 (1.34–1.55)	1.23 (0.98–1.47)	0.90	0.91 (0.87–0.95)
	Can	0.97 (0.91–1.03)	2.40 (2.00–2.79)	1.00 (0.99–1.02)	0.80 (0.77–0.83)	1.21 (0.90–1.51)
	Pac	0.80 (0.60–1.00)	3.57 (3.42–3.72)	1.57 (1.42–1.72)	1.92 (1.67–2.18)	—
	Gem	0.98 (0.93–1.03)	1.42 (1.18–1.65)	2.04 (1.82–2.23)	0.90	—
Pac+	Erl	0.84 (0.67–1.00)	1.58 (1.47–1.68)	0.76 (0.64–0.88)	0.55 (0.44–0.64)	—
	Lap	1.06 (0.89-1.24)	1.28 (1.11–1.44)	0.88 (0.84–0.93)	0.94 (0.83–1.05)	—
	Sap	0.77 (0.56–0.98)	0.70 (0.63–0.76)	0.87 (0.85–0.89)	0.84 (0.77–0.90)	—
	Afa	1.30 (0.97–1.63)	0.93 (0.86–1.01)	0.69 (0.63–0.76)	0.57 (0.52–0.62)	—
	Can	1.05 (0.71–1.39)	0.94 (0.76–1.11)	0.62 (0.48–0.76)	0.70 (0.60–0.79)	—
	Gem	3.61 (3.27–3.96)	1.54 (1.31–1.78)	1.32 (1.12–1.52)	1.56 (1.32–1.81)	—
Gem+	Erl	0.45 (0.38–0.52)	1.57 (1.06–2.09)	3.06 (2.56–3.55)	0.73 (0.71–0.75)	—
	Lap	1.00 (0.83–1.18)	2.07 (1.65–2.50)	3.62 (3.17–4.07)	1.29 (1.25–1.32)	—
	Sap	0.58 (0.56–0.60)	1.70 (1.28–2.12)	2.59 (1.71–3.46)	1.14 (1.07–1.21)	—
	Afa	1.07 (0.94–1.20)	1.33	2.24 (2.13–2.36)	0.81 (0.75–0.87)	—
	Can	1.62 (1.43–1.81)	2.34 (1.65–3.03)	2.83 (2.29–3.38)	1.17 (1.01–1.34)	—

Each value is the mean of triplicate samples from two independent experiments. Combination Index values < 0.9 = synergy; > 1.1 = antagonism; 0.9–1.1 = additive.

NVP = NVP-AEW541; Das = Dasatinib; Criz = Crizotinib; Pac = Paclitaxel; Gem = Gemcitabine; Erl = Erlotinib; Lap = Lapatinib; Sap = Sapitinib; Afa = Afatinib; Can = Canertinib; Das = Dasatinib; Criz = Crizotinib; Pac = Paclitaxel; Gem = Gemcitabine.

### No statistically significant relationship between EGFR, HER2 or HER3 expression and response to most HER-family TKIs

Linear regression was carried out to assess whether there was any correlation between sensitivity to treatment with our panel of agents and expression level of HER RTKs. No significant correlation was identified between expression of EGFR, HER2 or HER3 with the agents tested (data not shown) apart from canertinib with HER2 (*p* = *0.005*, R_2_ = 0.818). HER4 was not tested as levels were low in all cell lines.

### Irreversible HER-family TKIs are more effective at inhibiting phosphorylation of HER-family receptors and downstream signalling molecules

**Figure 5:**
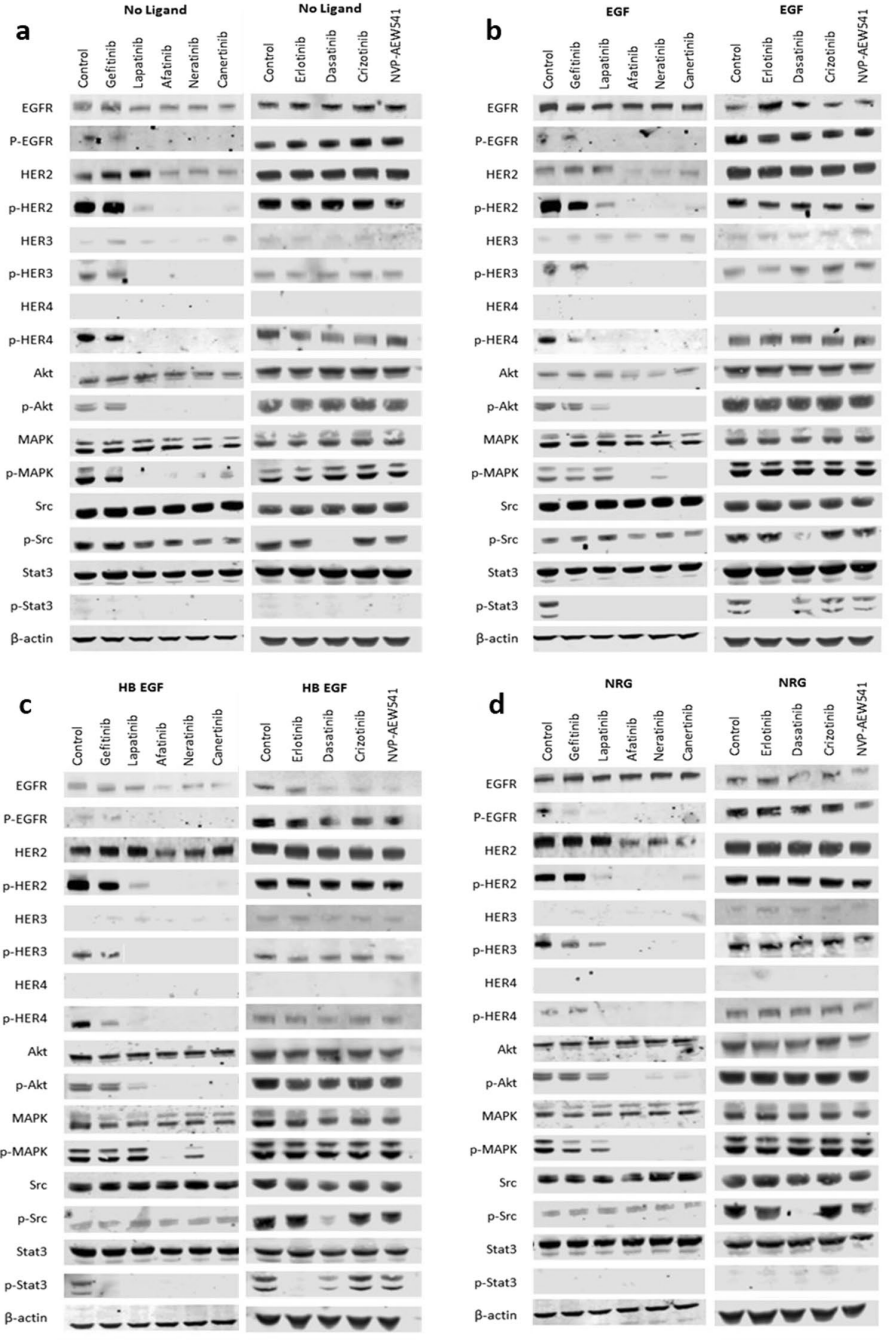
Effects of tyrosine kinase inhibitors on SKBr3 cell lines in the presence or absence of ligands. (**a**) No ligand. (**b**) EGF. (**c**) HB-EGF. (**d**) NRG. Cells were grown in 10% FBS DMEM until nearly confluent, then treated overnight with TKIs at 0.5% FBS DMEM overnight. The next day the cells were treated with 50 nM ligand for exactly 15 minutes before being lysed (with the exception of the control set, which was not treated with any ligand). Proteins were separated using SDS-page electrophoresis, transferred to PVDF membrane and the probed with primary and secondary antibodies.

SKBr3 cells were treated with various agents together with HER-family ligands EGF, HB-EGF and NRG (Fig. 5). The HER-family members were all constitutively active (phosphorylated) in SKBr3 cells in the presence or absence of ligands. Phosphorylation of HER-family members was inhibited by all HER-inhibitors, with afatinib and neratinib having the most pronounced effects, followed closely by canertinib. The reversible dual-inhibitor, lapatinib, inhibited phosphorylation to a lesser degree than the irreversible drugs, but considerably more than either of the EGFR-inhibitors, gefitinib or erlotinib, (Fig. 5). These results were consistent with the anti-proliferative effects of these agents in our growth control studies. Similar effects were observed in the phosphorylation of Akt, apart from in the presence of NRG, where afatinib had a superior inhibitory effect compared with neratinib and canertinib. MAPK phosphorylation was also inhibited in a similar pattern by HER-TKIs, though neratinib appeared to have an inferior inhibitory effect in terms of MAPK phosphorylation when cells were treated with EGF and HB-EGF. Phosphorylation of Stat3 (a transcription factor downstream of activated HER-family receptors) only occurred in the presence of EGF and HB-EGF ligands, but was strongly inhibited by all HER-TKIs (Fig. 5). Phosphorylation of HER family members, Akt and MAPK were generally unaffected by dasatinib, crizotinib or NVP-AEW541, the EGF and HB-EGF induced phosphorylation of Stat3 was slightly inhibited by dasatinib. Finally, of all the agents studied, only treatment with datatinib was highly effective at inhibiting both the constitutive and ligand-induced phosphorylation of Src in SKBr3 cells (Fig. 5).

### Western blot analysis shows low HER2 and Src expression in MDA-MB-453

**Figure 6:**
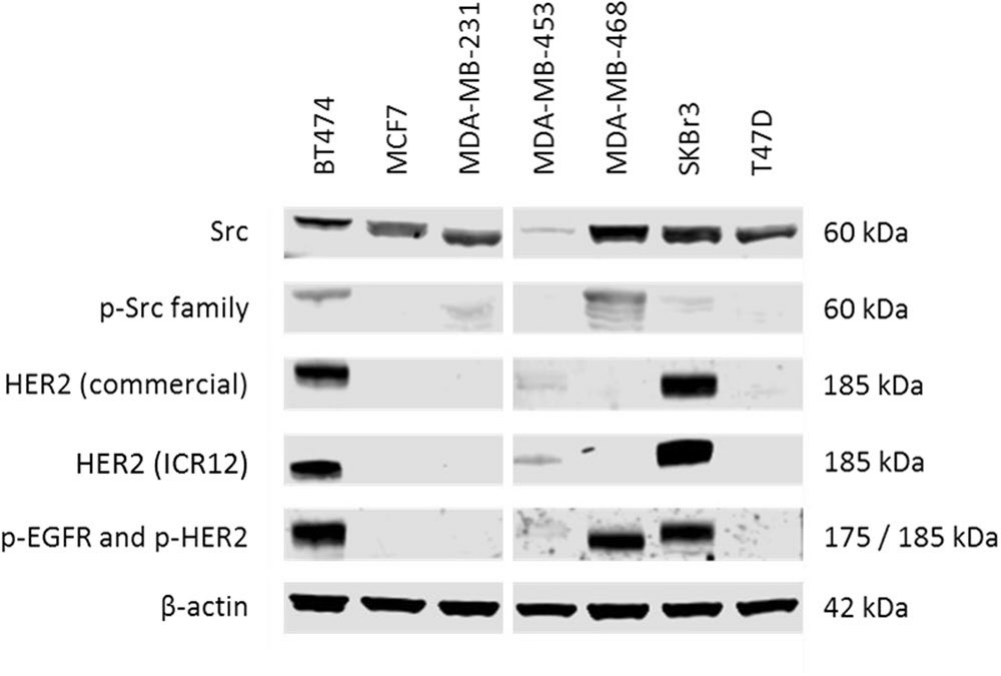
Western blot analysis of tyrosine kinase expression and phosphorylation in untreated breast cancer cell lines. Cell lines were grown to near-confluence in 10% FBS DMEM, washed in PBS and lysed. Proteins were separated using SDS-page electrophoresis, transferred to PVDF membrane and probed with antibodies targeting proteins of interest, including Src, phospho-Src, HER2 and phospho-HER2. The commercial anti-HER2 antibody (Cell Signaling Technology Inc) targeted the intercellular domain of HER2, while our in-house developed anti-HER2 antibody ICR12.27 targeted the extracellular domain. Negligible differences were observed between these two. Phospho-EGFR and phospho-HER2 were detected using the phospho-Src antibody, which had specific cross-reactivity with phosphorylated receptor tyrosine kinases.

Western blot was used to detect expression levels of Src, phospho-Src, HER2, phospho-EGFR and phospho-HER2 (Fig. 6) in our panel of breast cancer cell lines and the results were compared with those of flow cytometry. Src expression was present in all cell lines, though it was notably lower in MDA-MB-453. Phospho-Src was detectable in MDA-MB-468, BT474, SKBr3 and MDA-MB-231, but not in MDA-MB-453. HER2 had very high expression in BT474 and SKBr3, but, contrary to our flow cytometry results, there was very low expression in MDA-MB-453. Targeting of HER2’s intracellular domain, using commercially available anti-HER2 antibodies, versus mAb ICR12, targeting HER2’s extracellular domain, resulted in no significant difference in band strength. Expression of phospho-HER2 was also very low in MDA-MB-453 compared with BT474 and SKBr3 (Fig. 6).

### HER-family inhibitors, NVP-AEW541 and dasatinib target cells in G0/G1 phase, crizotinib, paclitaxel and gemcitabine target cells in S or G2/M phases

Flow cytometry was used to determine the effects of selected tyrosine kinase inhibitors and cytotoxic drugs on the cell cycle distribution of SKBr3 cells. Treatment with the HER-family inhibitors, NVP-AEW541 and dasatinib increased the percentage of cells in the G0/G1 phase and decreased the percentage in S and G2/M phases (Table 4a). Treatment with crizotinib led to an increase in the percentage of cells in S, G2/M phases and sub-G0 phase, with a decrease in cells in G0/G1 phase (Table 4b). Treatment with paclitaxel and gemcitabine led to increased percentages of cells in S and sub-G0 phases and decreased percentages of cells in G0/G1 phase (Table 4b).

**Table 4:** Effect on cell cycle distribution of SKBr3 cells following treatment with various agents. (a) HER-family inhibitors, NVP-AEW-541 and Dasatinib; (b) Crizotinib, paclitaxel and gemcitabine.

a	Cell Cycle Phase (% of gated cells)			
Treatment	Sub-G0	G0/G1	S	G2/M
Control	6.27	57.96	13.06	20.81
Erlotinib	8.54	69.85	6.36	13.81
Lapatinib	6.46	69.27	6.72	16.31
Sapitinib	8.45	69.53	5.51	15.55
Afatinib	10.22	67.59	5.49	15.67
Canertinib	5.46	61.14	11.31	20.53
NVP-AEW541	20.25	58.33	8.00	11.74
Dasatinib	11.84	70.1	3.72	13.02
**b**	**Cell Cycle Phase (% of gated cells)**			
**Treatment**	**Sub-G0**	**G0/G1**	**S**	**G2/M**
Control	6.83	65.75	9.7	14.46
Crizotinib	31.98	13.11	13.21	34.89
Paclitaxel	12.67	57.87	12.74	14.5
Gemcitabine	47.91	9.00	24.35	13.07

### Irreversible TKIs most effective at inhibiting migration of MDA-MB-231 cells

**Figure 7:**
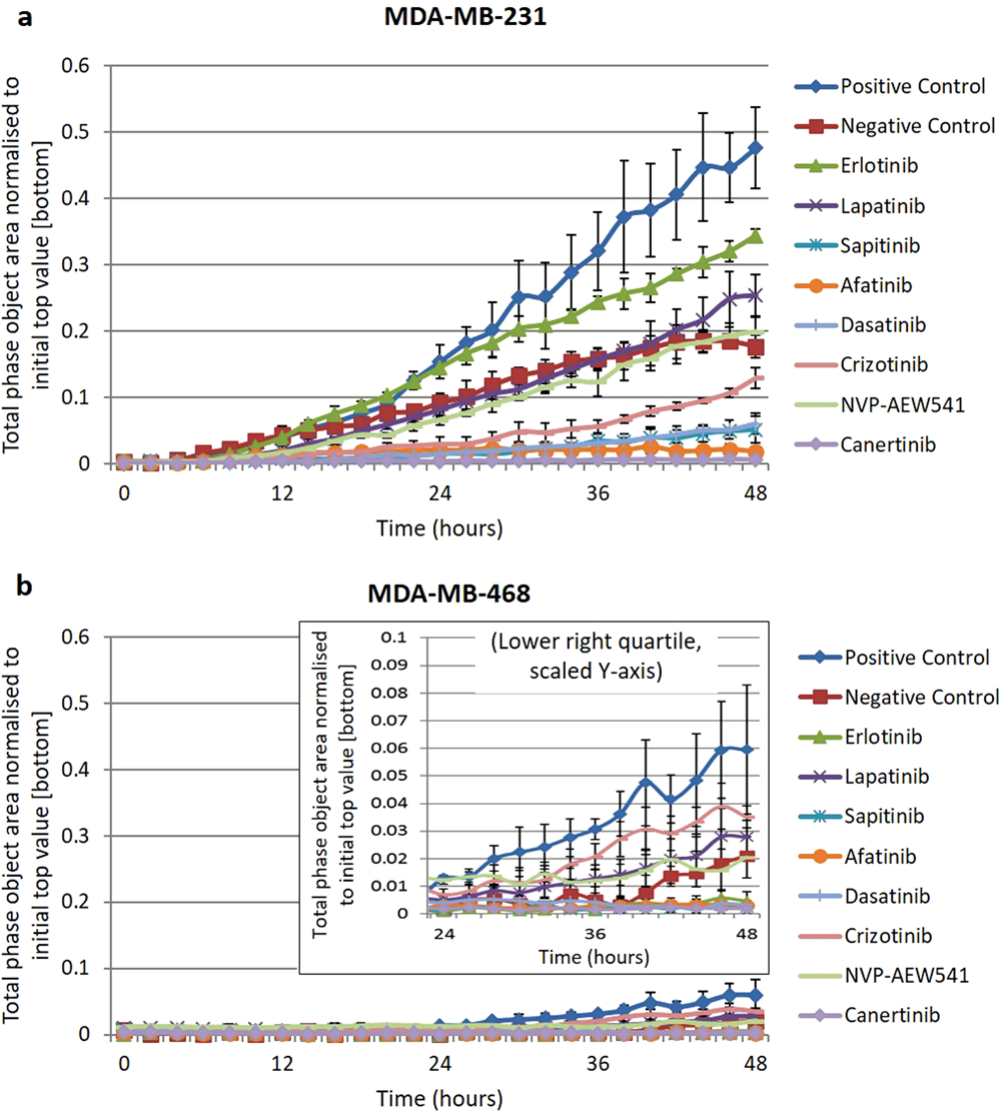
Effects of TKIs on migration of breast cancer cell lines. (**a**) MDA-MB-231. (**b**) MDA-MB-468. Cells were seeded into the top layer of a 96-well cell migration assay plate in 0.5% FBS DMEM together with TKIs at IC50 concentrations, while 10% FBS DMEM (chemo-attractant) was added to the bottom layer. Cells were incubated at 37 °C for 48 hours, with images taken using the Zoom Live-Cell Analysis System (Esso Bioscience) every 2 hours. MDA-MB-231 was highly motile, with significant migration observed after 12 hours. By contrast, MDA-MB-468 was much less motile, migrating only very slightly after 48 hours. All TKIs inhibited migration of MDA-MB-231, to varying degrees. Each point is a representative of the mean ± SD of triplicate samples.

The effects of treatment with selected agents at IC50 concentrations on cell lines SKBr3, MDA-MB-468 and MDA-MB-231 over a 48 hour period were investigated using the Incucyte Zoom Live-Cell Analysis System (Fig. 7). MDA-MB-231 had the highest level of migration in the positive control (no treatment, bottom layer supplemented with 10% FBS medium as chemo-attractant), while MDA-MB-468 had only very slight migration within 48 hours and none within 24 hours (Fig. 7). No significant migration of SKBr3 cells was observed within the 48 hour period (data not shown). After 24 hours, the irreversible pan-HER inhibitors and dasatinib had most effectively inhibited migration of MDA-MB-231 (Fig. 7). Migration of MDA-MB-468 was also inhibited, though these effects only became apparent after around 30 hours.

## Discussion

Overexpression of HER2 occurs in around 25% of human breast cancers cases and is usually associated with a relatively poor prognosis^11–14^. Currently, lapatinib is the only tyrosine kinase inhibitor approved for treatment of HER2 positive breast cancer, while the only other approved HER2 targeted therapies are the humanised antibodies trastuzumab and pertuzumab, and the antibody-drug conjugate Ado-trastuzumab-DM1 (T-DM1)^15, 35, 36^. While lapatinib significantly improves survival in HER2 + breast cancer patients, not all patients respond to treatment and many that do end up relapsing with acquired resistance to the initial treatment^15, 18, 20, 37^. Therefore, it is imperative to develop more effective therapeutic approaches and to discover novel biomarkers for more reliable screening of breast cancer patients who will benefit from available therapies.

Lapatinib is a dual-HER-targeting TKI and works by competing with ATP binding to the kinase domain of RTKs EGFR and HER2, thus preventing phosphorylation and downstream-signalling. However, this process is reversible and inevitable dissociation from the receptor is thought to be a contributing factor to resistance. Second-generation irreversible TKIs, such as neratinib, afatinib and canertinib, were designed to counter this by forming stronger covalent bonds with cysteine residues of the kinase active site, inactivating the kinase permanently^38–41^. In this study, we found that the irreversible pan-HER-family inhibitors, particularly afatinib and neratinib, were generally more effective than the reversible dual/pan-HER-family inhibitors lapatinib and sapitinib, which were in turn generally more effective than the reversible EGFR specific inhibitors, erlotinib and gefitinib (Table 2). The superiority of afatinib over reversible EGFR inhibitors was also reported by Ioannou *et al*.^42^, who demonstrated the superior growth inhibitory effects of afatinib in a panel of seven human pancreatic cancer cell lines. Next, we examined the effect of various types of HER inhibitors on the phosphorylation of HER family members and downstream cell signalling proteins, and found the irreversible pan-HER inhibitors were also more effective than the reversible HER inhibitors in reducing phosphorylation of the HER-family members, as well as downstream proteins Akt and MAPK, in SKBr3 cells (Fig. 5). Stimulation of SKBr3 with ligands (EGF, HB-EGF or NRG) did not significantly increase phosphorylation of HER-family members, suggesting constitutive activity of these receptors in SKBr3, likely due to the overexpression of HER2 (the preferred hetero-dimerization partner)^43, 44^. This suggests that the co-targeting of other HER-family members alongside HER2 using pan-HER inhibitors may be a mechanism for improved activity.

Of the seven breast cancer cell lines examined in this study, BT474 and SKBr3 had the highest level of HER2 expression determined by flow cytometry (MFI values of 540.81 and 467.53, respectively), and were the most sensitive to treatment with HER-family inhibitors as single agents. However, MDA-MB-453, which also overexpressed HER2 (MFI of 315.56) was considerably more resistant. For example, BT474 and SKBr3 were around 35–40 times more sensitive to afatinib and neratinib than MDA-MB-453, despite HER2 expression only being 0.5–0.7 times higher in those cell lines, highlighting that HER2 expression alone may not be a reliable indicator of sensitivity to treatments. This is consistent with previous studies that have described MDA-MB-453 as a HER2 overexpressing, but “lapatinib-resistant” cell line^20, 45–48^. Interestingly, other studies reported inconsistent results for the status of HER2 expression in MDA-MB-453. For example, Subik *et al*.^49^, Vranic *et al*.^50^ and Lehmann *et al*.^51^ have described this cell line as being “triple-negative” or having low HER2 expression. Conversely, Lee *et al*.^52^ and Sharieh *et al*.^53^ describe MDA-MB-453 as being an HER2 overexpressing cell line. Some studies have also identified this cell line as HER2 overexpressing but lacking in HER2 gene amplification^54^. The reasons for these discrepancies are uncertain, though they may be due to the varying sensitivities of the methods used to determine HER2 expression, as well as the different thresholds which were set for defining “overexpression”^46, 55^.

When we compared the results of our Flow Cytometry with Western Blot data, MDA-MB-453 had very high expression of HER2 in the former, but only a faint HER2 band in the latter, at least in comparison to BT474 and SKBr3 (Fig. 6). A possible cause for this difference could have been the presence of truncated forms of HER2 in MDA-MB-453 cells, leading to lower sensitivity to some detection methods^56, 57^. However, using anti-HER2 antibodies targeting both the internal and external domains of HER2, we found that bands were equally weak in MDA-MB-453 cells compared with BT474 and SKBr3 cells (Fig. 6), suggesting that it was unlikely that structural aberrations in HER2 were contributing to the discrepancy between methods. The levels of phospho-HER2 in MDA-MB-453 were also found to be significantly lower (barely detectable) than those in BT474 and SKBr3 cells (Fig. 6). Interestingly, according to flow cytometry, MDA-MB-453 had the highest expression of HER3 in all our cell lines, around 3–4 times higher than the HER3 expression of BT474 and SKBr3 (Table 1). HER3 is able to trans-phosphorylate HER2 and the two have been shown to co-operatively regulate tumour cell growth, with increased HER3 expression having been implicated as conferring resistance against HER2 targeting therapies^58–60^. Moreover, as it lacks internal kinase domain activity, targeting HER3 with tyrosine kinase inhibitors is relatively ineffective, perhaps explaining why even our pan-HER inhibitors were unable to completely overcome resistance in this cell line^61, 62^. Co-targeting the extracellular domains of HER2 and HER3 (e.g. with a dimerization inhibiting antibody such as pertuzumab) may therefore be able to overcome this cell line’s resistance to other HER2 targeted agents^54, 63^. Indeed, the anti-HER2 effects of trastuzumab have been shown to work synergistically with the dimerization blocking abilities of pertuzumab in certain breast tumour types, both *in vitro* and in the clinical setting^64, 65^. Additionally, we found that MDA-MB-453 had by far the lowest expression of Src kinase of all our cell lines and no detectable phospho-Src. This is unusual, given that Src overexpression and phosphorylation is normally upregulated in conjunction with HER2 overexpression^30, 31, 66^. Interestingly, Belsches-Jablonski *et al*.^31^ found that, despite having barely detectable Src levels in Western blot analysis, MDA-MB-453 was one of the few cell lines to have detectable levels of stable HER2/Src complexes, while no such complexes were detected in BT474 or SKBr3. This study proposed co-operative interactions between Src, HER2 and HER3 to promote cell growth and survival. Moreover, Src activation has been reported to participate in resistance to treatment with anti-HER2 antibody trastuzumab and poor prognosis in patients with HER2 positive breast cancer^67^. As a result, we examined the effect of treatment with combinations of the Src inhibitor dasatinib and HER-family inhibitors on the growth of MDA-MB-453 cells (Table 3). These combinations produced synergistic effects, suggesting that Src may contribute to resistance of this cell line to treatment with various forms of HER2-targeted agents, including reversible and irreversible small molecule HER TKIs. Further investigation is warranted on the therapeutic potential of HER inhibitors when combined with dasatinib, particulary in HER2 + breast cancer cells which are insensitive to HER inhibitors.

Interestingly, MDA-MB-453 and MDA-MB-231 were the only cells in our panel to harbour *K-Ras* mutations^50, 68^. MDA-MB-231 was highly resistant to HER-family TKIs, despite having moderate expression of HER2 and the second highest expression of EGFR. *K-Ras* mutation has been implicated as a potential contributor of resistance to HER-family targeted therapy, particularly in colorectal cancer^69, 70^, a mechanism also alluded to by Ioannou *et al*.^71^ who noted that, in a panel of pancreatic cell lines, the most sensitive to inhibition by HER-family inhibitors was the only one carrying a wild-type *K-Ras* gene. As EGFR and HER2 hetero-dimerise and have highly interrelated signalling pathways, and the dual and pan-HER inhibitors used in this study target both EGFR and HER-2, any effect of k-Ras mutations on EGFR sensitivity to these agents may have an effect on HER2 signalling. However, the direct effects of k-Ras mutation on HER2 in breast cancer are currently unclear, and warrant further investigation.

As explained earlier, in some studies the aberrant expression and activation of other receptor tyrosine kinase and downstream cell signalling molecules (e.g. IGF-1R, c-Met, Src) have been shown to co-operate with HER family members to drive tumour growth and to confer resistance to therapy including treatment with HER inhibitors^23–26, 31, 32^. The effects of a selection of agents targeting different tyrosine kinases and interfering with different stages of the cell cycle were therefore tested in combination on the growth of the HER2 overexpressing cell lines BT474, SKBr3 and MDA-MB-453, the EGFR overexpressing MDA-MB-468, and the low HER-family expressing MCF7.

In our study, we found that the IGF-1R inhibitor NVP-AEW541 combined with HER-family inhibitors had mainly synergistic effects in MCF7 and MDA-MB-468. The synergistic effect of co-targeting of the EGFR and IGF-1R systems in MDA-MB-468 may be explained by high and moderate levels of expression of EGFR and IGF-1R respectively (Table 1). MCF7 cells had the highest level of IGF-1R expression but had relatively low expression of HER-family members. In another recent study, Chakraborty *et al*.^72^ have reported that treatment of MCF-7 cells with a combination of an IGF-1R mAb and the HER2 targeting agents neratinib and trastuzumab resulted in synergistic growth inhibition of these breast cancer cells, supporting the need for further investigations on the therapeutic potential of co-targeting IGF-1R and HER family members in breast cancer.

We found that the combination of dasatinib with HER-family inhibitors had synergistic effects in MDA-MB-468 and MDA-MB-453, and mixed results in BT474 (Table 3). Both MDA-MB-468 and BT474 had the highest expressions of HER-family members (EGFR and HER2, respectively) and the highest levels of p-Src in our panel, possibly explaining the synergistic effects in these cell lines (Table 1, Fig. 6). In contrast, while MDA-MB-453 was HER2 positive, it was found to be Src negative according to our Western blot studies presented in Fig. 6. However, as discussed earlier, it has been proposed that stable HER2/Src complexes may be present in this cell line^31^, which may explain the synergistic effects of co-targeting both molecules in this cell line.

Finally, we found that the combination of the c-Met/ALK inhibitor crizotinib with HER-family inhibitors had generally synergistic effects in MCF7 and MDA-MB-468, and mixed results in MDA-MB-453 (Table 3). Interestingly, while MDA-MB-468 had the highest levels of both c-Met and EGFR, which could explain these synergistic effects, MCF-7 cells had very low levels of both EGFR and C-Met (Table 1). Further investigations are therefore warranted to determine the mechanisms by which the same combination of HER-family inhibitors and crizotinib produced synergistic effect in MCF7 cells.

We also examined the anti-tumour activities of the TKIs when used in combination with different cytotoxic drugs. While the two chemotherapeutic drugs inhibited growth at different stages of the cell cycle (G2/M, S phases) to the HER-family inhibitors (G0/G1 phase) (Table 4), co-targeting did not necessarily lead to synergistic effects, even when HER-family members were overexpressed (Table 3). Of the breast cancer cell lines in our panel, MDA-MB-231 has been shown to be highly motile and metastatic^73, 74^. Indeed, of the three breasts cancer cell lines used in our migration assay (MDA-MB-468, SKBr3 and MDA-MB-231), only MDA-MB-231 cells migrated significantly within 24 hours (Fig. 7). While MDA-MB-231’s proliferation was relatively resistant to treatment with the HER-family TKIs (Table 2), its migration was inhibited more effectively by the pan-HER inhibitors canertinib, afatinib and sapitinib, as well as dasatinib (Fig. 7). This is consistent with our growth control assay, in which dasatinib was also the most effective agent for inhibiting the growth of MDA-MB-231 cells (IC50 = 9 nM, Table 2). Our results suggest that while some of these agents may not have much anti-proliferative effect on MDA-MB-231, they may still have some clinical value due to their ability to inhibit migration, a key feature of metastatic cancer.

## Conclusions

Our results show that second-generation irreversible inhibitors of HER-family members are generally more effective than reversible HER-family TKIs at inhibiting growth, downstream signalling and migration of breast cancer cells. We have also demonstrated that combining HER-family inhibitors with NVP-AEW541, dasatinib or crizotinib may have superior effects compared with treatment as single agents in certain breast cancer subtypes. Further preclinical and clinical investigations are warranted to determine the therapeutic benefit of such drugs in combinations and for the identification of more reliable companion diagnostic tests for the selection of a more specific population of breast cancer patients who would benefit from such therapeutic interventions, particularly given the poor reliability of individual tyrosine kinase expression levels in determining response to treatments.

## Methods

### Tumour cell lines

A panel of seven human breast cancer cell lines was used in this study, including new batches of BT474 and MDA-MB-231, which were purchased from ATCC/LGC, and MDA-MB-468, SKBr3, MDA-MB-453, MCF7 and T47D which were provided as previously described^9, 75^, and certified by the LGC Cell line Authentication Service (Teddingtron, UK). All cell lines were grown in Dulbecco’s Modified Eagle’s Medium (Sigma-Aldrich, Gillingham, UK) supplemented with 10% Bovine Serum (heat inactivated) (PAA Laboratories, Yeovil, UK) and antibiotics: penicillin (50 units per ml), streptomycin (0.05 mg ml^−1^) and neomycin (0.1 mg ml^−1^) (Sigma-Aldrich), and routinely cultured at 37 °C in a 5% CO_2_ humidified atmosphere.

### Tyrosine kinase inhibitors and other reagents

Erlotinib, NVP-AEW541 and afatinib were kindly provided by OSI Pharmaceuticals (Farmingdale, USA), Novartis (Basel, Switzerland) and Boehringer Ingleheim (Vienna, Austria), respectively. Paclitaxel and gemcitabine were acquired from Sigma-Aldrich (Dorset, UK) and Healthcare at Home (UK), respectively, while Iressa (gefitinib) and crizotinib were purchased from Tocris (Avonmouth, UK), respectively. Lapatinib, sapitinib, canertinib, neratinib, imatinib and dasatinib were all acquired from Selleckchem (USA).

Primary and secondary antibodies for flow cytometry were purchased from R&D Systems (Abingdon, UK), while the secondary FITC-conjugated rabbit anti-mouse mAb STAR9B was purchased from AbD Seroctec (Kidlington, UK). Primary antibodies for western blot were obtained from Cell Signaling Technology Inc. (Massachusetts, USA), apart from the anti-EGFR antibody F4, which was obtained from Sigma-Aldrich, while secondary antibodies were purchased from Li-Cor Inc (Nebraska, USA).

### Cell growth studies

The effect of TKIs and chemotherapy drugs on the growth of human breast cancer cells was determined using the Sulforhodamine B (SRB; Sigma-Aldrich, UK) colorimetric assay, as previously described^42^. The concentrations required to inhibited 50% of cell growth (IC_50_) by each agent were calculated using non-linear least squares curve fitting (four parameter analysis, log (inhibitor) vs response, variable slope) using Gen5 software (Biotek, UK). The effects of combining selected agents was also assessed using the combination index (CI) described by Chou and Talalay^76^. Agents were mixed at 4 x their IC_50_ followed by eight doubling dilutions and their effects determined using the SRB assay. Median effect analysis was then performed using the Calcusyn software (Biosoft, Cambridge, UK) to determine CI, where < 0.9 indicated a synergistic effect, 0.9–1.1 indicated an additive effect and > 1.1 indicated an antagonistic effect.

### Cell surface expression of receptor tyrosine kinases

The cell surface expression of HER family members, IGFR, c-Met and ALK on our panel of breast cancer cell lines was determined by flow cytometry, as described previously^42^. Approximately 1 × 10^6^ cells were suspended in 1 mL 2% FBS medium in 1.5 ml Eppendorf and incubated by rotation for 1 hour at 4 °C with medium alone (negative control) or medium containing primary antibody at a concentration of 10 µg/mL. Cells were then washed in PBS three times by centrifugation at 246 × g, re-suspended in 1 mL 2% FBS medium and incubated with FITC-conjugated rabbit anti-mouse IgG secondary antibody (STAR9B; AbD Serotec) for a further 1 hour by rotation at 4 °C. Cells were washed again three times via centrifugation and finally re-suspended in 0.5 mL FACSFlow buffer (Becton Dickinson Ltd, UK). Cells were run through a BD FACSCalibur flow cytometer (Becton Dickinson Ltd), where a minimum of 10,000 events were recorded by excitation with an argon laser (488 nm) using the FL-1 detector (525 nm), and then analysed using the CellQuest Pro software (Becton Dickinson Ltd).

### Assessment of relationship between growth factor receptor expression level and response to agents

Linear regression was performed using SPSS (IBM) to assess the relationship between expression of growth factor receptors and sensitivity to our panel of inhibitors and other agents. *P* < 0.05 was considered statistically significant.

### Cell-cycle distribution analysis

The effect of various agents, including HER inhibitors, on the cell-cycle distribution of breast cancer cell line SKBr3 was investigated using flow cytometry, as described previously^42^. Approximately 2.5 × 10^5^ cells were seeded to T-25 flasks containing 10 mL of 2% FBS growth medium together with the various agents at ~IC75 (as determined by growth control assay) or control medium (untreated) and incubated at 37  °C (5% CO_2_) until the control flask was almost confluent. Cells were then harvested by trypsinisation and pooled with their respective supernatants. Cells were washed three times in cold PBS by centrifugation at 264 g for 5 minutes. After the third wash the cell pellet was re-suspended in 200 µL cold PBS, fixed by the addition of 70% ethanol and left overnight at 4 °C. Cells were then collected by centrifugation at 264 g for 5 minutes, washed once with PBS, and incubated with PI/RNAse buffer (Becton Dickinson Ltd) at room temperature for 35 minutes. Cells were run through a BD FACSCalibur flow cytometer (Becton Dickinson Ltd), where a minimum of 10,000 events were recorded by excitation with an argon laser (488 nm) using the FL-3 detector (620 nm), and then analysed using the CellQuest Pro software (Becton Dickinson Ltd).

### Western blot

The effect of various agents, including HER family targeting TKIs, on downstream signalling molecules of SKBr3 was investigated using western blot analysis^42^. Cells were grown to near confluence in four 6-well plates in 5 mL of 10% FBS growth medium. Cells were washed with 5 mL of PBS and incubated for 24 h with 400 nM of HER inhibitors or control (untreated), mirrored in each of the four plates. Following incubation with inhibitors, cells were incubated in the presence or absence of various ligands for 15 minutes. Cells were then washed with PBS and lysed using 400 µL lysis buffer (Invitrogen, Paisley, UK) containing protease inhibitor cocktail (Sigma-Aldrich). Lysates were homogenised using a syringe and needle and heated to 90 °C for 5 minutes. Samples were loaded at 30 µg into 4–12% Bis-Tris gels (Invitrogen) and separated using the XCell II Surelock Mini-Cell system (Invitrogen) and transferred onto Immobilon-FL PVDF membranes (Millipore, Watford, UK) using XCell II Mini-Cell Blot Module kit (Invitrogen). The membranes were then probed with various antibodies purchased from Cell Signalling Technology UK, apart from the F4 anti-EGFR mouse antibody which was purchased from Sigma-Aldrich. Membranes were further probed with IRDye secondary antibodies (Li-Cor Inc) and signals detected using the Li-Cor Oddysey CLx (Li-Cor Inc).

Western blot analysis was also used to detect Src and HER2 expression in all cell lines. HER2 was probed using a commercial antibody (Cell Signaling Technology Inc) targeting the intracellular domain and ICR12 targeting the extracellular domain^77^ in order to investigate potential structural aberrations in the receptor (see MDA-MB-453 comments above). Phospho-EGFR and phospho-HER2 were detected using a phospho-Src family antibody (Cell Signaling Technology Inc), which specifically cross-reacts with phosphorylated receptor tyrosine kinases.

### Effects of selected inhibitors on cell migration

The effect of selected tyrosine kinase inhibitors on cell migration was determined using the Incucyte Zoom Live-Cell Analysis System (Essen Bioscience), as described previously^78^. The EGFR overexpressing cell line MDA-MB-468, the HER2 overexpressing cell line SKBr3, and the highly-aggressive, triple-negative cell line MDA-MB-231 were selected for this assay. 1,000 cells were seeded into the top layer of a 96-well ClearView plate (Essen Bioscience) together with IC50 concentrations of inhibitors in 0.5% FBS DMEM. Following 30 minutes incubation (37 °C, 5% CO_2_), DMEM supplemented with 10% FBS (serving as the chemo-attractant) was added to the bottom layer of each well (apart from negative controls, which contained 0.5% FBS DMEM). The plate was placed into the Incucyte system which was set to take images every 2 hours. The Incucyte Chemotaxis software (Essen Bioscience) was then used to analyse the collected data.

### Data Availability

All datasets generated during and/or analysed during the current study are available from the corresponding author on reasonable request.

### Availability of data and material

The datasets during and/or analysed during the current study available from the corresponding author on reasonable request.
